# Delineating the Origins of *Vibrio parahaemolyticus* Isolated from Outbreaks of Acute Hepatopancreatic Necrosis Disease in Asia by the Use of Whole Genome Sequencing

**DOI:** 10.3389/fmicb.2017.02354

**Published:** 2017-11-28

**Authors:** Songzhe Fu, Huiqin Tian, Dawei Wei, Xiaojun Zhang, Ying Liu

**Affiliations:** ^1^College of Marine Technology and Environment, Dalian Ocean University, Dalian, China; ^2^Nanchang Center for Disease Control and Prevention, Nanchang, China; ^3^Institute of Oceanology, Chinese Academy of Sciences, Qingdao, China; ^4^College of Life Science, Northwest Agriculture and Forestry University, Yangling, China; ^5^College of Animal Science and Technology, Yangzhou University, Yangzhou, China; ^6^Nantong R&D Center, Chinese Academy of Sciences, Nantong, China

**Keywords:** AHPND, *Vibrio parahaemolyticus*, SNPs, whole genome sequencing, pVA-1 like plasmid

## Abstract

Acute hepatopancreatic necrosis disease (AHPND) is an emerging penaeid shrimp disease caused by *Vibrio parahaemolyticus*. Although *V. parahaemolyticus* has been isolated and sequenced from several Asia countries, the epidemiological links among the AHPND outbreaks in different locations remain unclear. In this study, we sequenced the genomes of nine strains isolated in China between 2014 and 2016 from four sampling sites in three provinces. Analysis of single nucleotide polymorphisms (SNPs) among the nine isolates yielded an average of 35,519 SNPs per isolate, ranging from 35,001 SNPs to 35,889 SNPs relative to the reference genome FDA_R31. To capture the genetic diversity of *V. parahaemolyticus* in Asia and Mexico, 93 published genomes were included in the analysis. Phylogenetic analysis divided the 102 isolates into 5 clades from I to V, with the majority belonging to Clade I and Clade II. There were at least 12 independent AHPND related clones. The results indicated that the clones recovered from AHPND affected shrimps in Asia were genomically distinct in various locations and there are no epidemiological links between Asian and Mexico outbreaks. Core genome analysis of pVA-1-like plasmid sequences from *V. parahaemolyticus* revealed that the AHPND-associated plasmids were also genetically diverse. Homology analysis of the publicly available microbial genomes showed that the conjugative transfer gene clusters of the plasmids in AHPND-causing strains were found in 27 *V. parahaemolyticus* strains and several other *Vibrio* sp. from 10 countries including five strains isolated prior to the first identification of AHPND outbreak, indicating that the backbone of AHPND- associated plasmid was widely distributed around the globe. In conclusion, at least 11 origins of AHPND outbreaks were identified; as AHPND-causing plasmid is widely distributed globally, prevention strategies for AHPND need to focus on microbial management in the aquaculture system and establishing ecological friendly aquaculture practices instead of detection of plasmid alone. However, more strains from other Asia countries as well as Mexico need to be included for whole genome sequencing (WGS) for reconstruction of the global transmission and the spread patterns of AHPND.

## Introduction

Acute hepatopancreatic necrosis disease (AHPND) is a recently emerged penaeid shrimp disease, which causes a pale and atrophied hepatopancreas (HP) together with an empty stomach and midgut (Tran et al., 2013). Since it was first found in the outbreaks of China in 2009 (Flegel, 2012), it has been subsequently found in Vietnam since 2010 (Tran et al., 2013), and in Malaysia and Thailand in 2011 and 2012, respectively (Kondo et al., 2014). It reached Mexico in early 2013 (Nunan et al., 2014), and was subsequently found in South America in 2016 (Restrepo et al., 2016). The loss of shrimp production caused by AHPND in the shrimp farming industry was estimated at more than $1 billion per year globally (FAO, 2013). Tran et al. (2013) suggested that the causative agent of AHPND was a specific strain of *Vibrio parahaemolyticus*. Lee et al. (2015) further confirmed that a 70-kb plasmid (pVA-1) carrying binary toxin genes *pirAB* in *V. parahaemolyticus* is responsible for AHPND symptoms. *V. parahaemolyticus* lives in warm estuarine and marine environments and distributes throughout the world (Lopez-Joven et al., 2015; Xie et al., 2016). As an important foodborne pathogen, *V. parahaemolyticus* is frequently isolated from seafood and became the leading cause of acute gastroenteritis worldwide (Letchumanan et al., 2015a,b). However, there is no molecular epidemiology study conducted for investigating the origins of AHPND, which left a gap in the understanding of the long-term transmission of AHPND related *V. parahaemolyticus*.

Recently, whole genome sequencing (WGS) has been successfully employed to identify and trace the bacterial outbreaks in aquaculture (Bayliss et al., 2017). Hossain et al. (2014) found that virulent *Aeromonas hydrophila* strains isolated from serial outbreaks of catfish farmes in the United States were phylogenetically related to the ones found in Chinese carp, which provided solid evidence to confirm their epidemiological links. WGS has also shown its advantages over other traditional pathogen-typing methods, such as Multilocus sequence typing (MLST), as it presented a higher resolution in typing the isolates from the same sequence types (STs) (Gonzalez-Escalona et al., 2017). As WGS utilise of all single nucleotide polymorphisms (SNPs) identified from the genomes, it helped to confirm the epidemiological links of outbreak isolates with higher typing resolution (Didelot et al., 2015). Although AHPND related *V. parahaemolyticus* has been isolated from several Asian countries, the epidemiological links among the outbreaks in different locations remained largely unclear.

The questions we aim to address are classical questions of epidemiology: does the sequential order of outbreaks indicate the transmission route of AHPND and does the first identification of AHPND in China necessarily indicate that the origin of AHPND? To date, there are over 10 AHPND related *V. parahaemolyticus* genomes sequenced (Gomez-Gil et al., 2014; Gomez-Jimenez et al., 2014; Kondo et al., 2014; Yang et al., 2014). However, their genomic evolutionary relationships have not yet been revealed. To fully characterize the epidemiological links among the isolates in different regions, in this study, we recovered and sequenced the isolates from shrimps from four regions in China. Analysis of nine isolates sequenced in this study together with publicly available 93 genomes revealed the genomic diversity of *V. parahaemolyticus* and multiple origins of AHPND outbreaks. Subsequently, the phylogenetic relationship among the plasmids was also analyzed to reveal their evolutionary relationship. To date, this is the first comparative genomic analysis of AHPND related *V. parahaemolyticus*.

To avoid confusion over the terminologies used, pVA-1 like plasmid was defined as a plasmid with over 40% coverage with AHPND-causing plasmid pVA-1. *V. parahaemolyticus* strains that carried pVA-1 like plasmid and were isolated from diseased shrimp, was designated as AHPND related isolates, while other *V. parahaemolyticus* strains were referred as non-AHPND related isolates.

## Materials and methods

### Sampling of diseased shrimp and sediments

To cover the genomic diversity of the representative *V. parahaemolyticus* in the coastal regions of China, we searched the publicly available genomes in the Genbank and found that current *V. parahaemolyticus* genomes covered seven coastal provinces and regions in China including Liaoning, Shandong, Shanghai, Guangdong, Taiwan, Guangxi and Hainan but not available for Jiangsu, Zhejiang and Fujian provinces. Therefore, diseased shrimp (*Litopenaeus vannamei*) specimens were collected from four local shrimp farms in Ganyu (Jiangsu), Qidong (Jiangsu), Hangzhou (Zhejiang), and Zhangpu (Fujian) from 2014 to 2016. The farms in Hangzhou, Rudong and Zhangpu experienced typical AHPND outbreaks (Supplementary Figure 1), while typical shrimp vibriosis symptom (red body disease) was observed in the farms from Ganyu and Rudong.

### Isolation of *V. parahaemolyticus*

The HP of shrimp was aseptically removed from diseased shrimp, and disaggregated, streaked onto thiosulfate citrate bile salts (TCBS) agar plates and incubated at 37°C for 24 h as described previously (Tran et al., 2013). Individual green colonies were identified and sub-cultured in tryptic soy broth (TSB) and incubated at 37°C for 24 h. The bacterial solution was restreaked on tryptic soy agar (TSA) plates to obtain pure isolates. For bacterial identification, isolates were PCR-amplified with 16S-23S rRNA intergenic spacers -specific primers VINTF (5′-TGGGGTGAAGTCGTAACAAGG-3′) and VINTR (5′-TCCTTCATCGCCTCTGACTG-3′) (Maeda et al., 2000). The PCR products were analyzed by 1% agarose gel electrophoresis followed by addition of SYBR-Green and visualization using an ultraviolet (UV) transilluminator. All of the isolates were stored at 25% glycerol stocks at −80°C.

### Confirmation of the causative agent for the outbreak

To test if *V. parahaemolyticus* isolated from shrimp can cause the disease for a healthy, susceptible shrimp with the same symptoms of AHPND or red body disease, infection tests were conducted. Before the experiment, shrimps (*Litopenaeus vannamei*) were acclimated for 6 days with a mean weight of 1.1 ± 0.4 g and 46 ± 5 mm in length and were fed twice a day. Ten percent of the shrimps were randomly selected and their HP was analyzed by standard microbiological analysis to determine if they were pathogens- free as described by Zorrilla et al. (2003). The infection test was conducted using juvenile shrimp in 20 L aquaculture tanks, with 20 shrimps in each tank. The experiment consisted of nine experimental groups and one group without the inoculation of *V. parahaemolyticus* as control, with three tanks for each group. Each overnight culture of *V. parahaemolyticus* was washed with 0.85% NaCl twice and suspended in sterilized seawater. Subsequently, it was inoculated in three tanks respectively and achieved the final concentration of 5.3 × 10^6^ Colony-Forming Units (CFU) per ml. Juvenile shrimps were then exposed to the inoculated *V. parahaemolyticus* for 5 days. The water temperature was controlled at 25°C by a heater during the experiment.

The HP of shrimp was collected within 24 h postinfection and disaggregated in a 0.85% NaCl solution. Subsequently, it was streaked on CHROMagar Vibrio agar (Haibo, Qingdao, China) and incubated at 37°C for 24 h, which further purified in TSA plates to obtain a pure culture. The isolation and molecular identification of pathogen were carried out as described above.

### Screening for the presence of pVA-1 like plasmid

The AP4 primers AP4-F1 (5′-ATGAGTAACAATATAAAACATGAAAC-3′), AP4-R1 (5′-ACGATTTCGACGTTCCCCAA-3′), AP4-F2 (5′-TTGAGAATACGGGACGTGGG-3′), and AP4-R2 (5′-GTTAGTCATGTGAGCACCTTC-3′) were used to amplify *pir*AB in nine sequenced strains as described by Dangtip et al. (2015). The pVA-1 plasmid kindly provided by Dr. Kallaya Sritunyalucksana was used as a positive control.

### Genome sequencing, *de novo* assembly and identification of single nucleotide polymorphisms (SNPs)

Nine strains were isolated and used for genome sequencing (Table 1). The phenol/chloroform method was used to extract genomic DNA from each strain as described previously (Pang et al., 2013). Genomic DNA was sequenced by Vazyme Biotech company (Nanjing) using the Illumina Genome Analyzer (Illumina) with 150 bp paired-end sequencing. To obtain the draft genomes, contigs from chromosomes and plasmids were *de novo* assembled using Velvet version 1.0.8 and VelvetOptimiser (Zerbino and Birney, 2008). Contigs were aligned to the reference *V. parahaemolyticus* genome FDA_R31 O1:K33 using progressiveMauve version 2.3.1 (Darling et al., 2010), as genome FDA_R31 is not genetically related to any outbreak strains. A stringent SNP calling was performed by a custom pipeline as described previously to guarantee that only genuine SNPs were included in the analysis (Chan et al., 2016). A custom script was used to extract SNPs according to the position on the reference genome. The SNPs in the public genomes were determined by using the NUCmer program “show-snps” in the MUMmer package (version 3.0) (Delcher et al., 2003).

**Table 1:** General features of *V. parahaemolyticus* strains used in this study.

**Strain No**.	**Source**	**Symptom**	**Location**	**Isolation date**
JN-8	Shrimp (*Litopenaeus vannamei*)	red body disease	Ganyu/Jiangsu	07/2014
JN-4	Shrimp (*Litopenaeus vannamei*)	red body disease	Ganyu/Jiangsu	07/2014
JN-7	Shrimp (*Litopenaeus vannamei*)	red body disease	Ganyu/Jiangsu	07/2014
LNM3-1	Shrimp (*Litopenaeus vannamei*)	AHPND	Rudong/Jiangsu	07/2015
NM-1	Shrimp (*Litopenaeus vannamei*)	red body disease	Ganyu/Jiangsu	07/2015
1930	Shrimp (*Litopenaeus vannamei*)	AHPND	Zhangpu/Fujian	08/2014
NM-3	Shrimp (*Litopenaeus vannamei*)	red body disease	Ganyu/Jiangsu	07/2014
DX-1	Shrimp (*Litopenaeus vannamei*)	red body disease	Rudong/Jiangsu	08/2014
HZ-7	Shrimp (*Litopenaeus vannamei*)	AHPND	Hangzhou/Zhejiang	06/2016

### Phylogenetic analysis of *V. parahaemolyticus* genomes and plasmid

The core genome content of *V. parahaemolyticus* genomes was obtained by respectively analyzing commonly shared regions of *V. parahaemolyticus* chromosomes using progressiveMauve (version 2.3.1) as described previously (Fu et al., 2015). The genes present in >90% of 102 isolates (Table S1) were defined as core genes as suggested by Ahmed et al. (2012) to minimize loss of genes from the core genome due to the stochastic errors in a few of low-coverage assemblies across this large dataset. The final alignment included 3,568 core genes with 3.68 M genome size. There were 4,600,901 total SNP positions in the alignment, of which 3,351,420 were variable among the *V. parahaemolyticus* strains. After the removal of recombinant SNPs (Supplementary Text, Supplementary Figure 2), the final SNPs alignment comprised of 3,331,653 sites. The phylogenetic tree based on non-recombinant core genome SNPs of *V. parahaemolyticus* was constructed using the Maximum Parsimony algorithms in PAUP 4.0 (Swofford, 1993). The global phylogeny was rooted using TUMSAT_H03_S5, which was shown to be most distant related to other *V. parahaemolyticus* lineage. The pVA-1 like plasmids from 14 AHPND related isolates were also analyzed by progressiveMauve 2.3.1 to obtain the plasmid core genome. To elucidate the genetic relationship among the plasmids, the phylogenetic tree based on core plasmid genome was inferred by PAUP 4.0 as well. The plasmid from strain ISF-54-12 was used as background genome.

### *In slico* multilocus sequence typing (MLST) typing

*In slico* MLST typing was performed by MLST 1.8 server from the Center for Genomic Epidemiology (https://cge.cbs.dtu.dk//services/MLST/) (Larsen et al., 2012).

### Gene content analysis

RAST was used to annotate the sequences from each draft genome (Aziz et al., 2008). These annotated genes were also grouped into functional categories. The presence of prophages from the sequenced strains was first screened using PHAST (Zhou et al., 2011). The prophages were confirmed by searching the presence of integrase characteristic of prophage genomes from annotated genomes. Antimicrobial resistance genes were identified using ResFinder (Zankari et al., 2012). Some unique genes were identified using progressiveMauve as they were not able to be aligned to FDA_R31. To confirm the presence of pVA-1-like plasmid, reads and contigs were mapped onto the reference pVA-1 plasmid genome (KP324996) in strain 3HP. Multiple plasmid comparisons were visualized using BRIG (version 0.95) (Alikhan et al., 2011).

### Definition of AHPND outbreak clone

Previous study suggested that the fastest molecular rate of *Vibrio Cholera* was around 3.3 SNPs/year/genome (Mutreja et al., 2011), while the fastest mutation rate of bacteria was up to 10 SNPs/year/genome (Loewe et al., 2003). These rates were used to infer the evolutionary timeframe for AHPND related isolates. The *V. parahaemolyticus* isolates had the pair-wise SNP difference < 10 SNPs per year were considered from the same outbreak clone. The formula was used to calculate mutational difference was: *N* = Nt/(y1-y2) (Jarvik et al., 2010), where Nt is the pair-wise SNP difference between two isolates, where y1 and y2 is the year of isolation for two isolates. If *N* > 10, two isolates were considered without the epidemiological link.

## Results

### Isolation of *V. parahaemolyticus* and their correlation with the outbreak

In total, nine *V. parahaemolyticus* strains were isolated from diseased shrimp (Table 1), two of which associated with AHPND outbreaks. PCR detection of *pirAB* genes showed that strain 1930 and HZ-7 were *pirAB* positive, while no amplicon was observed for LNM3-1. The remaining six isolates responsible for red body disease were all *pirAB* negative. To confirm if these isolates were the causative agent responsible for the outbreaks, infection tests were conducted. In the infection test, the diseased shrimps could only be found in the experimental groups inoculated with the nine strains. For the experiments infected with AHPND related *V. parahaemolyticus*, the shrimp stopped feeding immediately (after 2 h) in two experimental groups (1930 and HZ-7), and reached 100% mortality within 5 days (Table 2). For these two strains, the symptoms for the diseased shrimps included lethargy, empty stomach and empty midgut, which were consistent with what has been observed in the shrimp farms. However, shrimps infected with strain LNM3-1 did not exhibit any typical AHPND symptoms with only 10.0% mortality; shrimps from this group did not show any noticeable alteration in their HP.

**Table 2:** The pathogenicity of isolated *V. parahaemolyticus*.

**Experimental group**	**Presence of *pirAB***	**Final mortality (%)**	**Symptom**
JN-8	–	53.3	sluggish or vertical swimming; red pleopods; light-yellow cuirasses and gills
JN-4	–	63.3	sluggish or vertical swimming; red pleopods; light-yellow cuirasses and gills
JN-7	–	58.3	sluggish or vertical swimming; red pleopods; light-yellow cuirasses and gills
LNM3-1	–	10.0	sluggish swimming
NM-1	–	46.7	sluggish or vertical swimming; red pleopods; light-yellow cuirasses and gills
1930	+	100	lethargy and empty stomach and midgut
NM-3	–	45	sluggish or vertical swimming; red pleopods; light-yellow cuirasses and gills
DX-1	–	56.7	sluggish or vertical swimming; red pleopods; light-yellow cuirasses and gills
HZ-7	+	100	lethargy and empty stomach and midgut
Control	NA	5.8	NA

For the shrimp infected with strain JN-4, JN-8, JN-7, NM-1, NM-3 and DX-1, the diseased shrimps first displayed sluggish swimming or swimming vertically on day 1. Subsequently, shrimps stopped feeding, meanwhile the pleopods turned red on day 2. For the moribund shrimp, the whole body turned red and the cuirasses and gills became light-yellow. The mortality of shrimp for above six strains ranged from 43.3 to 63.3%. The bacteria re-isolated from moribund shrimps under aseptic conditions were all identified as *V. parahaemolyticus*, indicating that the isolated bacteria were the pathogens for the red body disease.

### General statistics of the genome sequencing

Nine *V. parahaemolyticus* strains were sequenced using MiSeq 150-bp paired-end sequencing in a multiplexed format. The average coverage depth for all genomes was 27, with the lowest coverage depth of 25 (Table 3). Percentage match of reads to reference strain FDA_R31 O1:K33 (CP006004/5, chromosome I and II) ranged from 92.1 to 95.3%. The reads were assembled *de novo*. The number of contigs ranged from 534 to 740, with an average of 660. HZ-7 had the largest genome size of 5.9 Mb, while JN-4 had the smallest (5.4 Mb). However, as a stringent SNP calling was employed to guarantee that only genuine SNPs were included (Chan et al., 2016), the high number of contigs did not affect the following phylogenetic analysis.

**Table 3:** General features of *V. parahaemolyticus*genomes sequenced in this study.

**Strain No**.	**N50**	**Contig Number**	**Total Length (bp)**	**Coverage**	**Percentage mapped to FDA_R31**	**No. of SNPs**	**Accession**
JN-8	326,217	617	5,446,784	25	92.1	35,889	SRX3093857
JN-4	326,084	697	5,430,509	25	92.5	35,886	SRX3093858
JN-7	252,980	598	5,568,867	31	95.3	35,203	SRX3093859
LNM3-1	298,847	639	5,634,305	27	93.8	35,679	SRX3093860
NM-1	498,800	534	5,412,507	26	93.2	35,013	SRX3093853
1930	256,936	717	5,790,989	26	92.4	35,750	SRX3093854
NM-3	498,810	687	5,427,265	28	93.7	35,001	SRX3093855
DX-1	315,589	716	5,679,608	27	92.5	35,505	SRX3093856
HZ-7	274,024	740	5,817,304	28	94.3	35,749	SRX3093852

### Phylogenetic relationship of *V. parahaemolyticus* genomes

A total of 102 *V. parahaemolyticus* isolates, including 93 publicly available genomes, were selected and analyzed by core genome SNP typing and *in slico* MLST typing. These strains represented a diverse geographical distribution of *V. parahaemolyticus*. Isolates were collected between 1951 and 2016 with the majority (92.3%) isolated from Asia, of which 48.5% from China.

**Figure 1:**
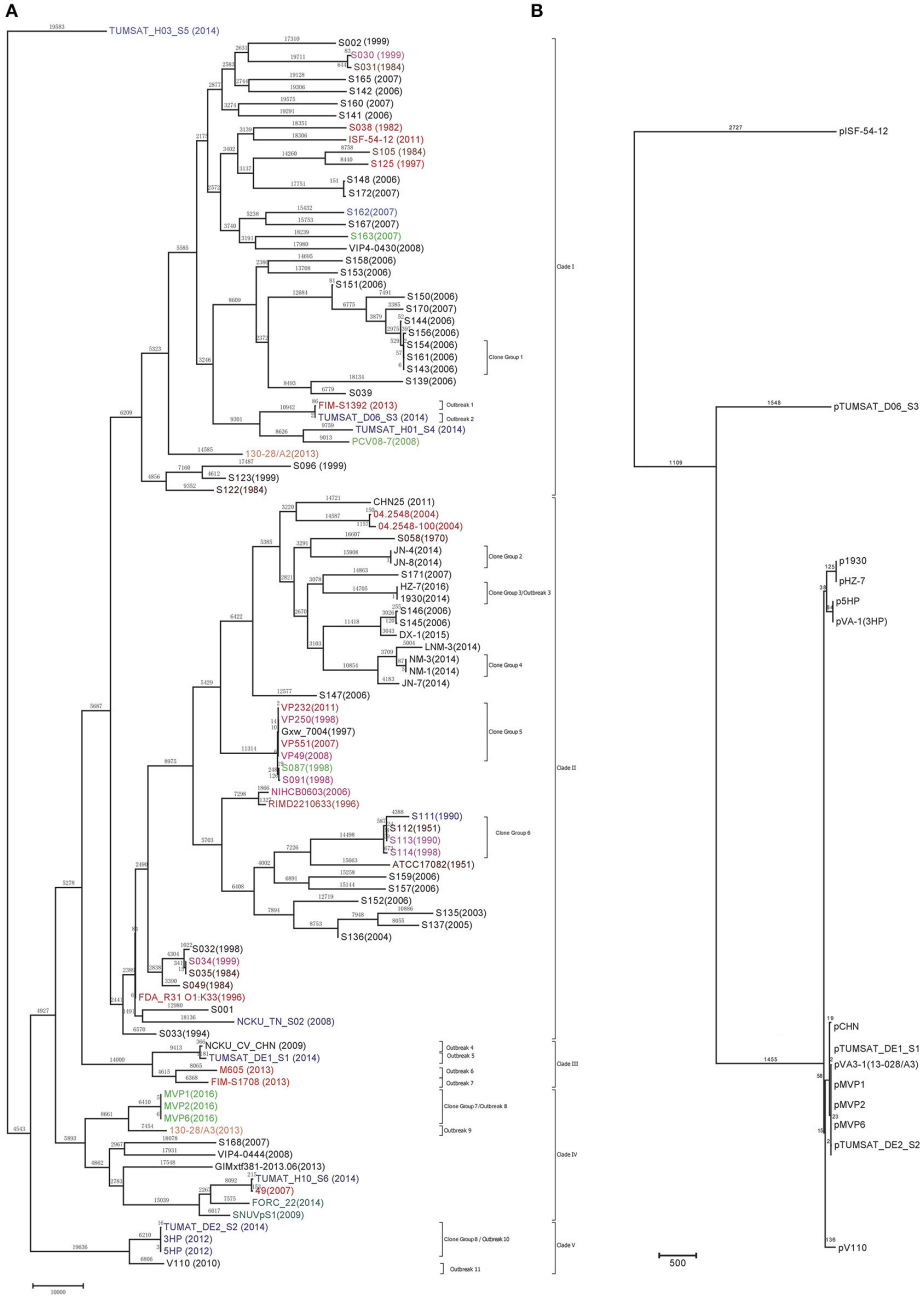
Maximum-parsimony tree of *V. parahaemolyticus* chromosomes **(A)** and pVA-1 like plasmids found in this study **(B)**. Homoplasy index (HI) is 0.09 for Maximum-parsimony tree of *V. parahaemolyticus* genomes, while HI is 0 for Maximum-parsimony tree of pVA-1 like plasmid. The possible AHPND related outbreak strains were indicated from outbreak one to twelve. The tree was rooted using strain TUMSAT_H03_S5. The number above the branches indicates the number of SNPs in *V. parahaemolyticus* chromosomes **(A)** and pVA-1 like plasmids **(B)**, respectively. The strains from different countries were indicated in various colors: Black, China; Red: Canada, USA and Mexico; Pink: India and Bangledash; Blue: Thailand; Light Green: Malaysia and Singapore; Orange: Vietnam; Dark Green: South Korea; Brown: Japan. The year of isolation was indicated in the bracket. The Maximum-parsimony tree of pVA-1 like plasmids was starched to match their corresponding chromosome genomes.

Maximum parsimony (MP) method was used to infer the phylogenetic tree using non-recombinant core genome SNPs. Only one MP tree was generated with Homoplasy Index (HI) of 0.09, indicating a low level of parallel changes. Amongst the 102 *V*. *parahaemolyticus* genomes, the strains were separated into five clades with 86 strains in two clades (Figure 1A). The most divergent strain was TUMSAT_H03_S5 which was used as an outgroup.

TUMSAT_H03_S5 had the largest number of strain-specific SNPs (24,126 SNPs), followed by S160 with 19,575 SNPs and S142 with 19,306 SNPs. Based on the definition of clone we defined in the previous section, there were eight clone groups identified in this study, some of which belong to AHPND related outbreak. For AHPND related isolates, results showed that they can be divided into at least 11 possible clones. We, therefore, assigned them into distinct outbreaks. Strain FIM-S1392- and TUMSAT_D06_S3 differing by 88 SNPs were denoted as Outbreak 1 and Outbreak 2, respectively. Outbreak 4 included strain 1930 and HZ-7 which were also highly clonal with 3 SNPs difference. TUMSAT_DE1_S1 (Thailand), NCKU_CV_CHN (China), M605 and FIM-S1708- represented Outbreak 3 to Outbreak 7 respectively, as strain-specific SNPs range from 366 to 8,065. MVP1, MVP2 and MVP6 isolated from Malaysia with maximum 6 SNPs difference were defined as Outbreak 8. Outbreak 9 included the strain 13-028/A3(Vietnam) with 7,454 strain-specific SNPs. 3HP, 5HP and TUMSAT_DE2_S2 which isolated from Thailand were grouped together as Outbreak 11 and separated by 19 SNPs. Strain V110 isolated from Hongkong in 2010 has 6,806 strain-specific SNPs, indicating that it belonged to an independent clone.

As conventional typing technique for *V. parahaemolyticus*, MLST typing also has shown the potentials to identify *V. parahaemolyticus* outbreaks and determine their sources (Gonzalez-Escalona et al., 2017). We also analyzed 102 genomes by *in silico* MLST typing to determine if MLST typing has sufficient typing resolution to differentiate outbreak clones. In total, 102 isolates were assigned into 53 STs by MLST typing including 9 unknown STs (Supplementary Figure 3). Compared with the MLST typing, genomic typing showed more precise resolution, as it divided 102 strains into 102 genome types, while many clone groups were assigned into the same STs by MLST typing. For instance, Clone Group 1 was assigned as ST-419, while Clone Group 5 belonged to ST-3. In addition, genomic typing resolved the phylogenetic relationships between the outbreak clones that not clearly evident from MLST typing. TUMSAT_DE1_S1 (Thailand) and NCKU_CV_CHN (China) both assigned into ST-114, but were clearly categorized into two outbreak clones by genomic typing. Interestingly, Clone Group 7 and 8 belonged to the new ST type 8 and ST970 respectively, which was also assigned as Outbreak 9 and 11, respectively.

### Phylogenetic relationship of pVA-1 like plasmids in the AHPND related isolates

Having shown that the *V. parahaemolyticus* from Asian AHPND outbreaks were not constituted by a single clone, we further investigated if the outbreaks were caused by the transmission of a conservative plasmid.

We assembled the plasmid genomes in AHPND related isolates including 5HP, TUMSAT06_DE2_S2, TUMSAT_D06_S3, V110, MVP1, MVP2, MVP6, CHN, M605, FIM-S1392-, FIM-S1708+ as well as strain 1930 and HZ-7 sequenced in this study. The plasmids were named as p5HP, pTUMSAT_DE1_S1, pTUMSAT_DE2_S2, pTUMSAT_D06_S3, pMVP1, pMVP2, pMVP6, pCHN, pM605, pFIM1392, and pFIM1708, p1930 and pHZ-7, respectively (Supplementary Figure 4A). The core genome of these plasmids was further analyzed to infer their phylogenetic relationship. However, the plasmids genome from three Mexico strains were excluded, as these genomes can only be partially recovered (Supplementary Figure 4B). A total size of 28 kb was found commonly shared by 14 plasmids (Table S2) and was used to construct a Maximum Parsimony tree (Figure 1B). This tree is unrooted as we cannot determine which plasmid arose earliest. Overall, the clustering of the plasmids also largely reflected the evolutionary relationship of the chromosomes. The plasmid pTUMSAT_D06_S3 belonged to an independent lineage, while the plasmids from HZ-7 and 1930 were located at another branch. The plasmids from AHPND related isolates in Clade III, Clade VI, and Clade V were clustered together. One exception is p5HP and pVA-1 which represented an independent cluster separated from pTUMSAT_DE1_S1, indicating that strains from outbreaks might acquire plasmids from different sources.

The plasmid sequences also showed a striking divergence, as SNP in the plasmid core genome ranged from 0 to 2,727 for the isolates found in different gographical regions. There was no SNP difference between p1930 and pHZ-7, which is consistent with the observation made from their chromosomes. pTUMSAT_DE1_S1, pTUMSAT_DE2_S2, pVPA3-1(13028-A3), pCHN, pMVP1, pMVP2, and pMVP6 showed little divergence as pair-wise SNP difference ranged from 0 to 23, while pV110 from a Hongkong isolate showed considerable divergence with 136 SNP difference. pVA-1 and p5HP formed another cluster but differed by 209 SNPs with p1930 and pHZ-7.

The plasmid pTUMSAT_D06_S3 formed another independent lineage, while pISF-54-12 was distantly related to other Asian plasmids. The comparative genomic analysis also showed that pISF-54-12 genome only had 95% identity with pVA-1 genome, which is the lowest identity in the genomes included in Figure 1B. Although the phylogenetic relationships of the plasmids from three Mexico strains were unknown, BLASTn results also showed that pM605, pFIM-1392-, and pFIM-17082+ only had 95, 94, and 89% identities against pVA-1 genome respectively, indicating they might be even more divergent than pISF-54-12.

**Figure 2:**
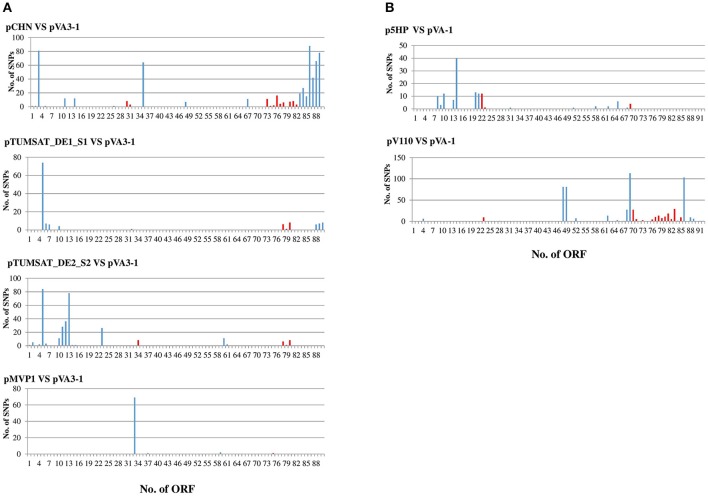
Pair-wise SNP comparison of isolates from the same plasmid cluster. pVA3-1 and pVA-1 were used as reference genome for pMVP1, pCHN, pTUMSAT_DE1_S1, and pTUMSAT_DE2_S2 **(A)**, and p5HP and pV110 **(B)** respectively. Blue bar indicates the SNP position in non-core genes of pVA-1 or pVA3-1, while red bar indicates the SNP position in the core genome of the plasmids. X-axial indicates the order of open reading frame (ORF) in pVA3-1 or pVA-1 genome.

Above results indicated that AHPND was possibly transmitted through plasmid transfer. However, as the core genome shrank largely from 70 to 28 kb, the SNPs in non-core genes might be neglected. Whether plasmids from the same cluster in Figure 1B belonged to the same plasmid clone remains unclear. To elucidate the genetic diversity of these plasmids in the non-core region, pair-wise SNP comparisons were conducted for p1930 and pHZ-7, p5HP and pVA-1, pV110 and pVA-1, pTUMSAT_DE1_S1 and pVA3-1(13028-A3), pTUMSAT_DE2_S2 and pVA3-1, pCHN and pVPA3-1, pMVP1 and pVA3-1. Results showed that p1930 and pHZ-7 only harbored one SNP difference, while no SNPs was found among pMVP1, pMVP2 and pMVP6, indicating that plasmids in both clusters might be respectively originated from the same clone. However, pTUMSAT_DE1_S1, pTUMSAT_DE2_S2, pCHN showed 128, 276 and 524 SNP difference in the non-core genes relative to pVPA3-1 (Figure 2A) respectively, suggesting that the plasmids in this cluster were also genetically diverse.

Interestingly, the sequence of pMVP1 was almost identical to pVA3-1, but harbored a hyper-variable hypothetical protein with 69 SNPs (position ranged from 25,960 to 25,367 relative to pVA3-1). Therefore, pMVP1 should also be excluded from the clones of pVA3-1.

p5HP also processed 115 SNPs in the non-core genes relative to pVA-1 despite their chromosome genome were genetically related, while pV110 has 373 non-core genes SNPs compared with pVA-1 (Figure 2B).

We also investigated the sequence variations of *pir*AB among the *pir*AB positive plasmids. Results showed that the sequence of *pirAB* was highly conservative among the plasmids, which showed 100% identity to all of the plasmids from *V. parahaemolyticus*, regardless of the origins of the isolates. The *pir*AB gene in the plasmid pVH from *V*. *owensii* also showed 100% identity, while the plasmid from *V*. *harveyi* had one SNP (Table S3), which was in agreement with a previous study (Xiao et al., 2017). The prophages were also variably presented in the sequenced genomes (Table S4).

### Presence of pVA-1 like plasmid in sequenced and public genomes

We further assembled the plasmids from remaining seven sequenced genomes and found that JN-8, JN-4, LNM3-1 and DX-1 also harbored the plasmid with partial homology with pVA-1 like plasmid (Supplementary Figure 4C).

To confirm if pVA-1 like plasmids were widely distributed globally, we used pVA-1 plasmid reference genome and blasted it into the microbial genome database. Results showed that at least 27 *V. parahaemolyticus* genomes harboured a complete or partial pVA-1 like plasmid (Table 4). Interestingly, most of the plasmids from these non-AHPND related isolates shared 34 kb backbone region (Conjugative transfer gene clusters), indicating they shared a common ancestor. However, they all lacked a 30 kb region. This region consists of three fragments. The first fragment ranging from 25 to 33 kb relative to pVA-1 position encoding genes for antirestriction protein, DNA primase and DNA binding protein, while the second fragment contains *pirAB* with two transposes in the left and right flanking regions (also called *pirAB*-Tn903 composite transposon in Xiao et al., 2017). The third region with 7.5 kb has 82% identity with an *Escherichia coli* genomic island QG7 (DQ499637) (Supplementary Figure 4D), indicating this region might be obtained in pVA-1 by a horizontal transfer event. The plasmids from three South Korea strains SNUVpS-1, FORC_22 and FORC_04 shared the same common region in pVA-1 plasmid (46% coverage/99% identity), but lacked *pirAB*-Tn903 composite transposon and downstream sequences (Table S5).

**Table 4:** Homologues shared across different plasmids.

**Strain name**	**Country**	**Source**	**Year**	**Size of plasmid (kb)**	**Coverage (%)**	**Identity (%)**	***pirAB***
***Vibrio parahaemolyticus***							
MVP1	Malaysia	Shrimp	2016	68	96	98	+
MVP2	Malaysia	Shrimp	2016	68	96	98	+
MVP6	Malaysia	Shrimp	2016	68	96	98	+
TUMSAT_D06_S3	Thailand	Shrimp	2014	65	91	97	+
3HP	Thailand	Shrimp	2012	51	72	99	+
NCKU_CV_CHN	China	Shrimp	2010	69	97	97	+
V110	China	Shrimp	2010	65	92	98	+
M605	Mexico	Shrimp	2013	65	57	95	+
TUMSAT_DE1_S1	Thailand	Shrimp	2014	66	79	98	+
TUMSAT_DE2_S2	Thailand	Shrimp	2014	70	80	98	+
13028-A3	Vietnam	Shrimp	2011	70	99	99	+
ISF-54-12	Canada	Imported shrimp	2011	86	54	95	+
FIM-S1392-	Mexico	Shrimp	2013	35	49	94	+
FIM-S1708+	Mexico	Shrimp	2013	34	29	89	+
S172	China	Environment	2007	33	46	95	–
S145	China		2007	26	37	95	–
GIMxtf381-2013.062013.06	China	Fish	2013	47	65	97	–
VPA-67	India: Andhra Pradesh	Shrimp	2013	69	46	95	–
JN-8	China	Shrimp	2014	110	30	95	–
JN-4	China	Shrimp	2014	110	30	95	–
LNM3-1	China	Shrimp	2015	64	66	95	–
1930	China	Shrimp	2012	69	97	98	+
DX-1	China	Shrimp	2013	91	5	97	–
HZ-7	China	Shrimp	2016	69	97	98	+
SNUVpS-1	South Korea	Crab	2009	65	50	94	–
FORC_22	South Korea	Crab Marinated in Soy Sauce	2014	110	32	94	–
FORC_04	South Korea	aquarium water	2014	64	65	94	–
***Vibrio harveyi***							
CUB2	Cuba: Varadero	Shore seawater	2014	26	37	98	–
KC13.17.5	Vietnam	Shrimp	2014	65	92	95	+
***Vibrio owensii***							
SH14	China	Shrimp	2013	70	99	99	+
***Vibrio campbellii***							
200612B	unknown	unknown	2006	18	25	98	–
051011G0092	Japan	seawater	2012	48	68	85	–

Besides, pVA-1 like plasmid were also identified in several other *Vibrio* sp. including *Vibrio owensii* strain SH14, *Vibrio harveyi* strain KC13.17.5, and *Vibrio Campbellii* 051011G, *Vibrio campbellii* 200612B (Supplementary Figure 4D).

## Discussion

### AHPND outbreaks have multiple origins

*Vibrio parahaemolyticus* is found to be widely present in the environments, including sediments, plankton, and aquatic animals (Su and Liu, 2007; Alipour et al., 2014). The pandemic *V. parahaemolyticus* caused by serotype O3:K6 and its serovariants were observed worldwide since 1996, which started in Southeast Asia and quickly spread to Europe and America (Nair et al., 2007). Previous research revealed that they all belong to a single clonal group (Okuda et al., 1997). To confirm if the AHPND related *V. parahaemolyticus* is also derived from a certain pandemic clone, in this study, we first analyzed the SNPs from all of publicly available AHPND related *V. parahaemolyticus* genomes as well as others isolated from seafood. Application of WGS and determination of SNPs in the core genome of the isolates clearly showed that AHPND related isolates were genetically diverse. Phylogenetic analysis also showed that the all AHPND related isolates could be clearly differentiated into distinct clusters each specific for different regions.

Therefore, 8-years lasted AHPND outbreaks in different regions can not contribute to a single pandemic clone. The phylogenetic analysis of the core genome of pVA-1 plasmid also showed considerable divergence among the pVA-1 like plasmids with at least three clusters. Therefore, we concluded that the AHPND epidemics were not caused by transmission of one genetically monomorphic lineage; most of the outbreaks occurred independently. Plasmid transfer was also not identified among the majority of AHPND outbreaks. However, we did observed the transmission of AHPND in nearby region as three and two strains were highly clonal in Malaysia and China, respectively. 3HP, 5HP, and TUMSAT_DE2_S2 from Thailand also probably came from the same origin.

### How AHPND outbreaks transmitted from asia to mexico remains unclear

Over the past several decades, Asian shrimps have been imported into the central Americas. In 2013, AHPND outbreaks were also identified in Mexico, which was thought to be originating from importing Asian shrimps. Although we found a Canada strain ISF-54-12 carried the pVA-1 plasmid, no Asian or Canadian isolates were clonally linked with the Mexico isolates. Therefore, the exact origin of Mexico AHPND outbreaks remains unknown. One possibility to explain this phenomenon is that we haven't sequenced and identified the Asian genomes that are clonally associated with Mexico strains.

However, three Mexico strains also showed a significant genome divergence with each other, indicating that there were at least three origins of AHPND in Mexico. Moreover, *in silico* bioinformatic analysis showed that both *pirAB* and the backbone of pVA-1 plasmid were also distributed in non- AHPND affected region including Cuba, India, South Korea and Japan.

There are three possible ways in which AHPND related isolates might have been introduced in Mexico: (i) transport of imported shrimp from Asia may have introduced the isolates which were clonal to M605, FIM-S17082+ or FIM-S3192- (to date, no such strain is found) (ii) the introduction of Asian shrimp into the Mexico may resulted in an introduction of an Asian *V. parahaemolyticus* that served as a pVA-1 plasmid-carrier and transferred the plasmid into other local isolates in Mexico (again, no such Asian strain is found) or (iii) the plasmids were widely distributed in the world, the practices of shrimp aquaculture facilitated the transmission of AHPND related *V. parahaemolyticus* among each region. Therefore, based on our analysis, the outbreaks in Mexico and Asian countries were likely independent events. Due to the limited number of accessible genomes, reconstruction of transmission route of AHPND was not warranted until extensive genomic sequencing of AHPND related *V. parahaemolyticus* in Asian countries and Mexico was conducted.

### The pVA-1 like plasmids were widely distributed worldwide

In our study, we identified at least 27 *V. parahaemolyticus* strains and five strains from other *Vibrio* sp. that harbored pVA1-like plasmids. These strains came from eight countries and at least five sources (fish, shrimp, crab, sediment, water). Previous study also supported this point which confirmed part of our results (Xiao et al., 2017). Interestingly, many isolates came from non-AHPND affected regions such as the South Korea (crab, water), India (shrimp), Japan and Cuba (seawater), indicating the ubiquitous distribution of the plasmid. Moreover, many isolates were obtained prior to the first outbreak of AHPND in late 2009, such as SNUVpS-1 (2009), S172 (2006), S145 (2006). Surprisingly, six out of nine sequenced strains harbored the pVA-1 like plasmids in this study, indicating a high prevalence of pVA-1 like plasmids in Jiangsu and Zhejiang provinces. One plasmid from LNM3-1 lack *pirAB* region although it was isolated from AHPND affected ponds. Therefore, it is likely that the pVA-1-like plasmid was widely distributed around the globe prior to the first outbreak of AHPND. Previous study suggested that there are multiple derivatives of the pVA-1 plasmid in *V*. owensii and other *Vibrio* sp. (Xiao et al., 2017). With more completed plasmid genomes becoming available, the evolution of pVA-1 like plasmid would be better delineated.

The gene organization of pVA1 showed that two transposes were located in both side of *pirAB*, further suggesting that *pirAB* may be frequently transferred among the *V. parahaemolyticus* or even other *Vibrio* sp. via transposition (Xiao et al., 2017), as *pirAB*-Tn903 composite transposon was found to be inserted into various plasmids at different sites. Results from Xiao et al. (2017) even suggested that *pirAB*-Tn903 composite transposon in the plasmid can be lost through *in vitro* passaging, further indicating the instability of the transposon. Above observations indicated that *pirAB*-Tn903 composite transposon might be also ubiquitously distributed in the environment, which leads to the stochastic insertion into the pVA1-like plasmid.

Given the wide-spread occurrence of *V. parahaemolyticus* with various lineages among different regions of China (Hongkong, Hainan, Jiangsu, Fujian and Zhejiang provinces) and reports of strains in South-eastern Asia in the years prior to the AHPND epidemic, the most likely scenario is that there were multiple pathogenic *V. parahaemolyticus* lineages distributed in different geographical locations, causing the AHPND independently due to the acquisition of the plasmid or *pirAB*, a few of which were introduced into the new locations due to clonal transmission.

Our results also have implications for long-term epidemiological surveillance of *V. parahaemolyticus*. Firstly, surveillance solely based on the epidemiological survey is likely to leave a gap for the long-term epidemiology of *V. parahaemolyticus* and WGS is likely to be a golden standard for diagnosis and outbreak investigation of AHPND in the near future. Secondly, current strategies which only focus on preventing the spread of AHPND by limiting the import of shrimps from Asian countries need a fundamental change. As the isolates that carried the backbone of pVA-1 like plasmid were widely distributed globally, adopting pathogen-free shrimp and the detection of *pirAB* alone before conducting the shrimp aquaculture is likely insufficient to guarantee the success of aquaculture. Detection of backbone of pVA-1 like plasmid would be also essential, as *pirAB*-negative plasmid also would be the recipient for *pirAB*-Tn903 composite transposon which can transfer among the *Vibrio*. sp (Dong et al., 2017; Xiao et al., 2017). Our results highlight that *pirAB* might only be one of key triggers for massive mortality. Shrimp monoculture system has been beset with devastating losses with infection and environment deterioration due to high stocking density. In such rearing pond, high concentration of shrimp feces and unconsumed feed promoted the rapid growth of *Vibrio* sp. Shrimps exposed to this environment over time would become susceptible to bacterial infections. Boonyawiwat et al. (2017) also found that increased stocking density contributed to an increased risk of AHPND infection, which demonstrated that effective disease control programs need to emanate from shrimp hatcheries. Given the fact that plasmid is ubiquitously distributed, future disease prevention studies need to focus on microbial management in the aquaculture system (De Schryver and Vadstein, 2014). Establishing ecological friendly aquaculture practices (such as Tilapia-Shrimp polyculture) and reducing the stocking density of shrimp would be essential for a sustainable shrimp aquaculture. In addition, our results also indicated that MLST has limited value for long-term epidemiological surveillance as frequent STs (such as ST3) dominate the endemic genotypes, which reduces the power of MLST for outbreak investigations. This study also confirmed that two isolates belonging to the same ST were divided into different outbreaks by WGS. While this study was underway, whole genome MLST was also developed which may provide a solution to overcome the pitfalls of traditional MLST (Gonzalez-Escalona et al., 2017).

To reconstruct the transmission route of *V. parahaemolyticus* isolates successfully, it depends on, not only typing them at a sufficient level of resolution, but also placing enough isolates with epidemiological information in the correct timeline. In this study, WGS provided more precisely typing solution, which corroborated the epidemiological investigation with suspected transmission events. One caveat of this study is that the isolates we selected may not recover the full transmission route of *V. parahaemolyticus* from the shrimp farms. Recent emergence of AHPND in South America also underscores the urgency of global cooperation to delineate the origins and transmission of AHPND related *V. parahaemolyticus* outbreaks by the use of WGS. Large-scale WGS of AHPND related isolates from China and Southeast Asian will be conducted in our future study.

## Conclusion

In summary, whole genomic analysis of *V. parahaemolyticus* genomes showed that there is no evidence to support that China served as a sole source of global pandemic of AHPND. Genomic typing resolved the phylogenetic relationships between the outbreak clones more precisely than MLST typing. The pVA-1 like plasmid (with or without *pirAB*) is widely distributed worldwide. This study provides a broader understanding of the global genomic diversity of AHPND related *V. parahaemolyticus*.

## Ethics statement

All shrimps in this study were handled in strict accordance with China legislation on scientific procedures on living animals. The protocol was approved by the ethics committee at Chinese Academy of Science (permit number: 20021101).

## Author contributions

Conceived and designed the experiments: SF and YL. Sampling and isolation of strains was performed by HT, DW, and XZ. HT performed the infection test. Data analysis and the draft of manuscript were performed by SF and YL. All authors approved the final version of the manuscript for submission.

### Conflict of interest statement

The authors declare that the research was conducted in the absence of any commercial or financial relationships that could be construed as a potential conflict of interest.
