# Supplementary material for: Delineating the Origins of Vibrio parahaemolyticus Isolated from Outbreaks of Acute Hepatopancreatic Necrosis Disease in Asia by the Use of Whole Genome Sequencing
Source: Front Microbiol. 2017 Nov 28;8:2354. doi: 10.3389/fmicb.2017.02354 (PMC5712426; doi:10.3389/fmicb.2017.02354)
Supplement: Supplementary file 2 [file Image1.pdf]

Supplementary Material

Delineating the origins of *Vibrio parahaemolyticus* isolated from outbreaks of acute hepatopancreatic necrosis disease in Asia by the use of whole genome sequencing

Songzhe Fu[1, 2] , Huiqin Tian[3], Dawei Wei[4], Xiaojun Zhang[5], Ying Liu[1, 6]

1. College of Marine Technology and Environment, Dalian Ocean University, Dalian, 116023, China
2. Nanchang Center for Disease Control and Prevention, Nanchang, 330038, China
3. Institute of Oceanology, Chinese Academy of Sciences, Qingdao, 266071, China
4. College of Life Science, Northwest Agriculture and Forestry University, Yangling, 712100, China
5. College of animal science and technology, Yangzhou University, Yangzhou, 225009, China
6. Nantong R&D Center, Chinese Academy of Sciences, Nantong, 226019, China

*** Correspondence:** Corresponding Author: E-mail: fusongzhe@hotmail.com

1 Supplementary text

Uneven role of recombination in *V. parahaemolyticus*

Recombination events were identified using RDP3 (Martin and Rybicki, 2000). Seven algorithms, including GENECONV, Bootscan, MaxChi, Chimaera, SiScan, original RDP and 3Seq were employed to detect recombinant signals, simultaneously. Recombinant segments were confirmed only if recombinant signals were detected by at least three algorithms for a particular sequence.

Results showed the relationship between high SNP density and recombination events. Overall, 201 kb region in the studied genomes were attributed to homologous recombination. There were 112 recombination events in 201 kb region which introduced 19,767 SNPs.

Most of the likely recombination sites were located within a 160 kb region (2,562,679–2,725,178 in chromosome I) which was surrounded around the O- and K-antigen encoding gene cluster (Supplementary Figure 2). Our results were consistent with a previous study which also found a high proportion of recombination regions in O- and K-antigen (Cui et al., 2013). Some of recombination were probably imported from other serovars of *V. parahaemolyticus* and led to serovar shift events.

Most of the recombination regions in the genome contained a high frequency of SNPs but there were also a few of long regions with low SNPs density. However, these recombination events were rare.

Prophage diversity of sequenced strains

The nine sequenced strains exhibited differences in prophages, plasmids, and antibiotic resistance genes profiles. In total, 14 prophages or prophage remnants, including VfO3K6, VCY_phi, 12B8, VFJ, 12B12 and VEJphi, were identified among the nine *V. parahaemolyticus* genomes (Table S4).

Reference

Cui, Y., Yang, X., Didelot, X., Guo, C., Li, D., Yan, Y., Zhang, Y., Yuan, Y., Yang, H., Wang, J., Wang, J., Song, Y., Zhou, D., Falush, D. and Yang, R. (2015) Epidemic clones, oceanic gene pools, and eco-ld in the free living marine pathogen *Vibrio parahaemolyticus*. Mol Biol Evol. 32, 1396-410. doi: 10.1093/molbev/msv009.

Martin, D. and Rybicki, E. (2000) RDP: detection of recombination amongst aligned sequences.Bioinformatics.16, 562-3.

2 Supplementary Figures

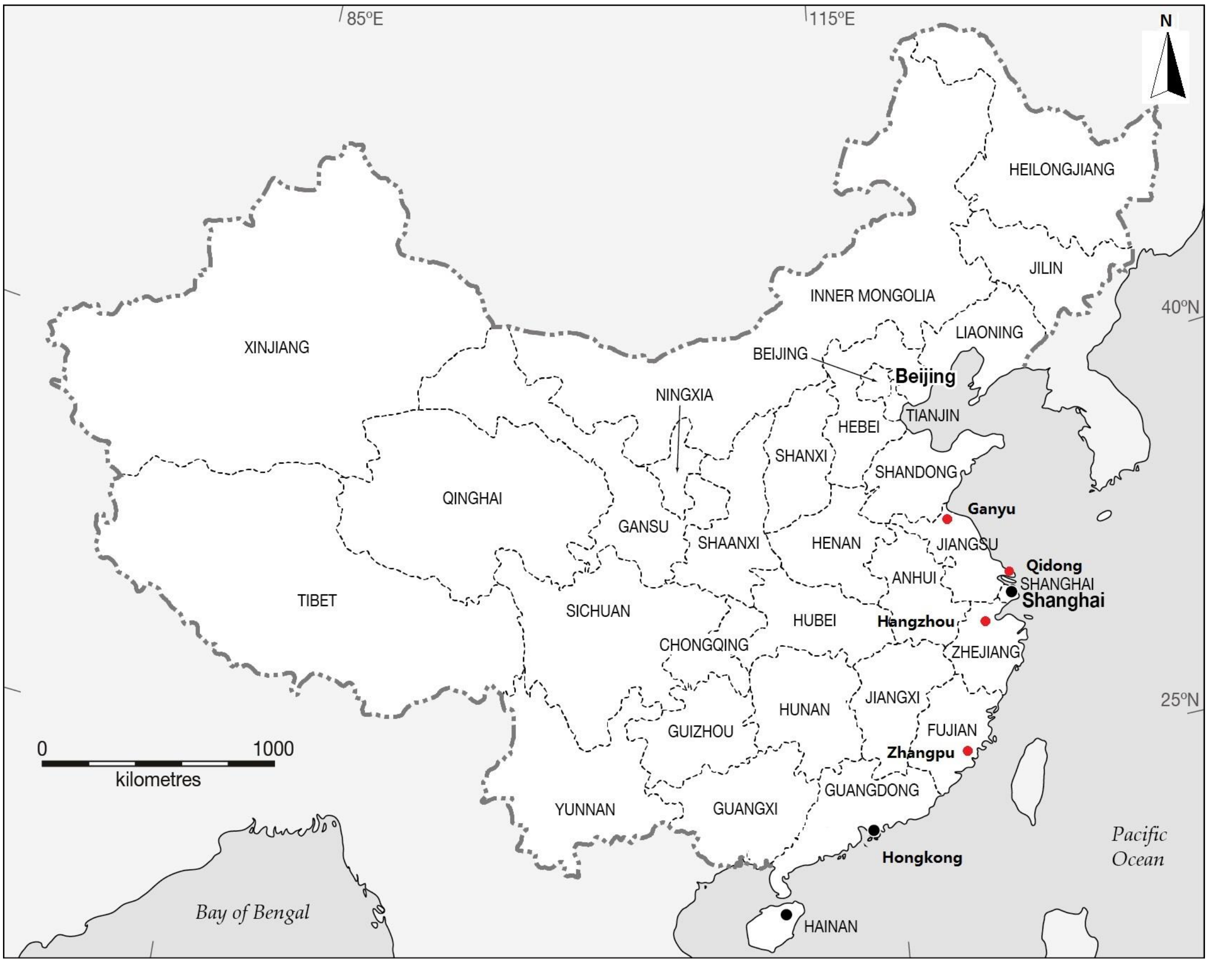

Supplementary Figure 1. Sampling sites of the shrimp farm in this study. Location of four sampled shrimp farm was indicated as red circle while the location of reported isolates with pVA1-like plasmid found in Genbank was indicated as black circle. The sampling sites were mapped by the ArcGIS Desktop 10.2 software.

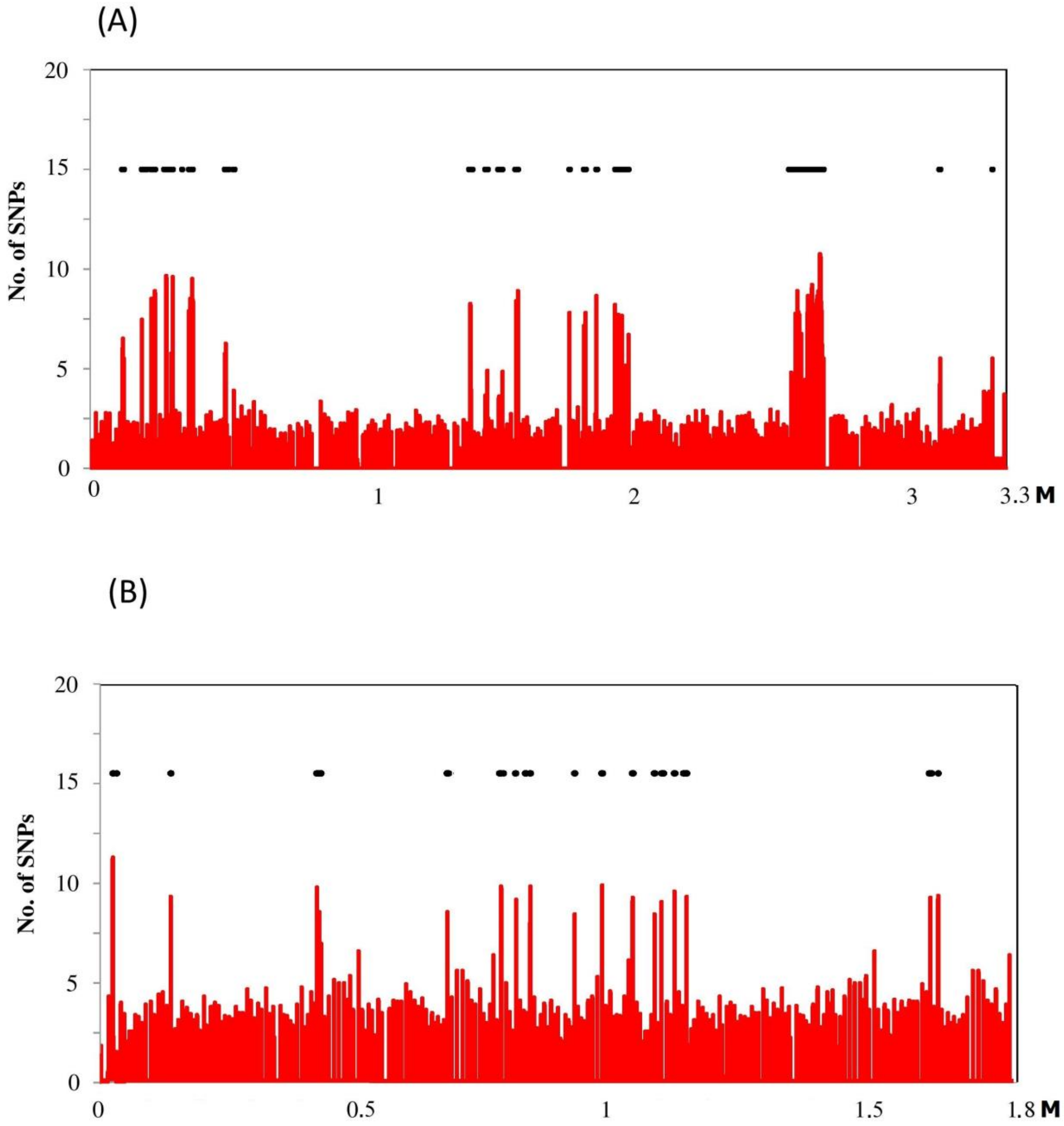

Supplementary Figure 2. Positions of recombinant regions in chromosome I (A) and II (B) based on the *V. parahaemolyticus* reference genome FDA_R31. The X-axis indicated the genomic position in chromosome I (A) and II (B). Recombinant segments are detected by RDP3 and indicated as horizontal black lines spanning regions. The red lines indicated the SNP density, which represented the average number of SNPs among 102 genomes counted within 10-kb windows across the chromosome.

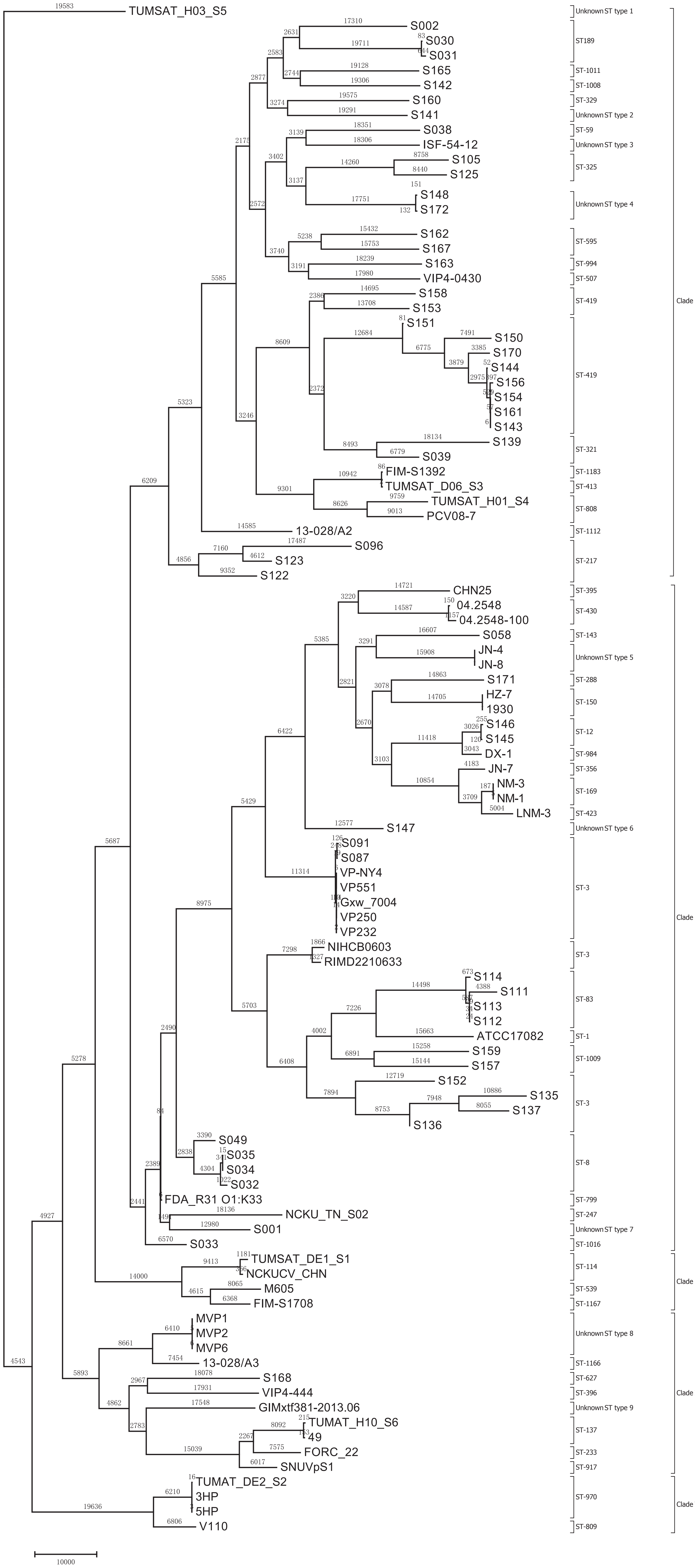

TUMSAT_H03_S5
S002
S030
S031
S165
S142
S160
S141
S038
ISF-54-12
S105
S125
S148
S172
S162
S167
S163
VIP4-0430
S158
S153
S151
S150
S170
S144
S156
S154
S161
S143
S139
S039
FIM-S1392
TUMSAT_D06_S3
TUMSAT_H01_S4
PCV08-7
13-028/A2
S096
S123
S122
CHN25
04.2548
04.2548-100
S058
JN-4
JN-8
S171
HZ-7
1930
S146
S145
DX-1
JN-7
NM-3
NM-1
LNM-3
S147
S091
S087
VP-NY4
VP551
Gxw_7004
VP250
VP232
NIHCB0603
RIMD2210633
S114
S111
S113
S112
ATCC17082
S159
S157
S152
S135
S137
S136
S049
S035
S034
S032
FDA_R31 O1:K33
NCKU_TN_S02
S001
S033
TUMSAT_DE1_S1
NCKUCV_CHN
M605
FIM-S1708
MVP1
MVP2
MVP6
13-028/A3
S168
VIP4-444
GIMxtf381-2013.06
TUMAT_H10_S6
49
FORC_22
SNUVpS1
TUMAT_DE2_S2
3HP
5HP
V110
10000
Unknown ST type 1
ST189
ST-1011
ST-1008
ST-329
Unknown ST type 2
ST-59
Unknown ST type 3
ST-325
Unknown ST type 4
ST-595
ST-994
ST-507
ST-419
ST-419
ST-321
ST-1183
ST-413
ST-808
ST-1112
ST-217
ST-395
ST-430
ST-143
Unknown ST type 5
ST-288
ST-150
ST-12
ST-984
ST-356
ST-169
ST-423
Unknown ST type 6
ST-3
ST-3
ST-83
ST-1
ST-1009
ST-3
ST-8
ST-799
ST-247
Unknown ST type 7
ST-1016
ST-114
ST-539
ST-1167
Unknown ST type 8
ST-1166
ST-627
ST-396
Unknown ST type 9
ST-137
ST-233
ST-917
ST-970
ST-809
Clade I
Clade II
Clade III
Clade IV
Clade V

Supplementary Figure 3. Comparsion of MLST typing with core genome typing. The STs were indicated in the right-hand side of Maximum-parsimony tree of *V. parahaemolyticus* genomes.

(A)

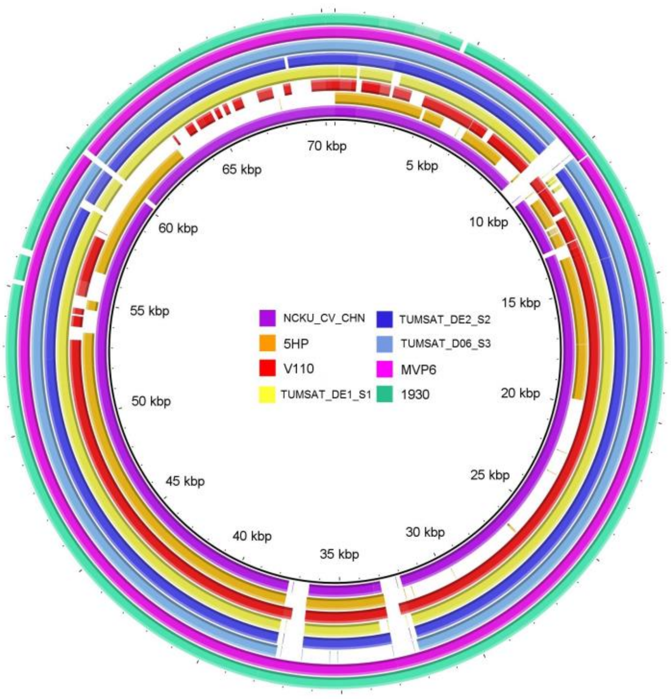
70 kbp
5 kbp
10 kbp
15 kbp
20 kbp
25 kbp
30 kbp
35 kbp
40 kbp
45 kbp
50 kbp
55 kbp
60 kbp
65 kbp
NCKU_CV_CHN
5HP
V110
TUMSAT_DE1_S1
TUMSAT_DE2_S2
TUMSAT_D06_S3
MVP6
1930

(B)

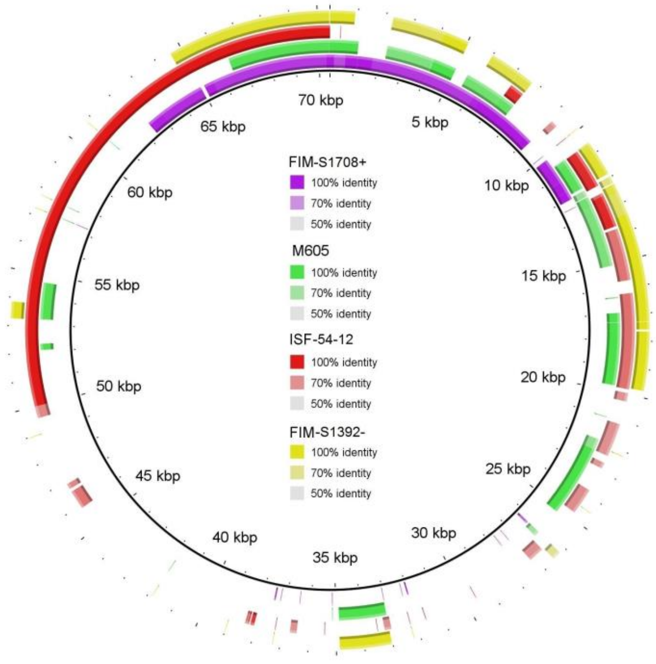
70 kbp
5 kbp
10 kbp
15 kbp
20 kbp
25 kbp
30 kbp
35 kbp
40 kbp
45 kbp
50 kbp
55 kbp
60 kbp
65 kbp
FIM-S1708+
100% identity
70% identity
50% identity
M605
100% identity
70% identity
50% identity
ISF-54-12
100% identity
70% identity
50% identity
FIM-S1392-
100% identity
70% identity
50% identity

(C)

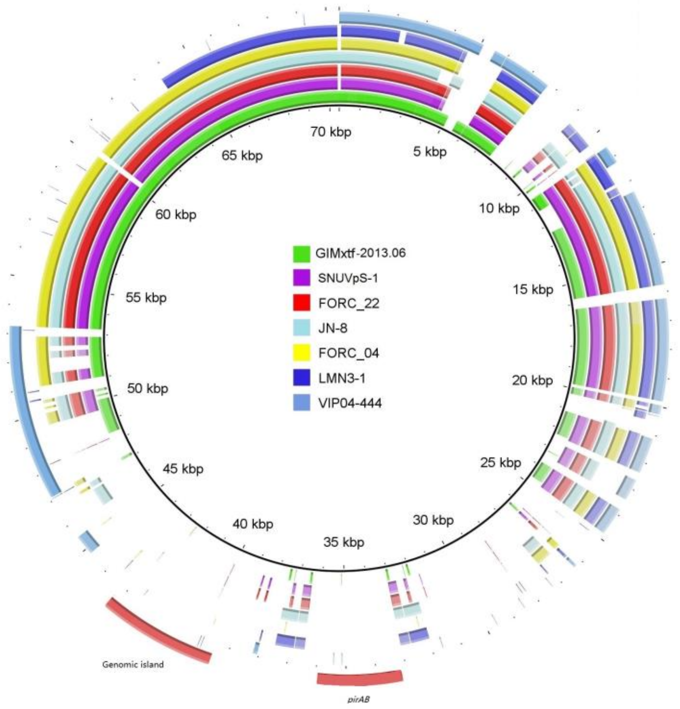

(D)

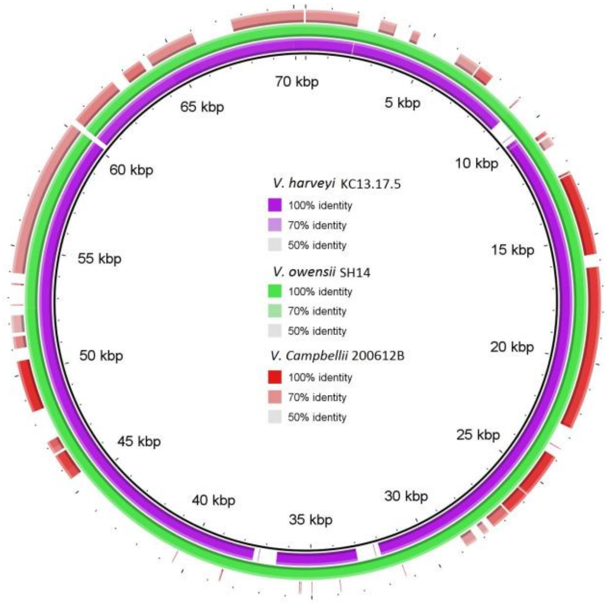

Supplementary Figure 4. Multiple genome comparisons using BRIG with pVA-1 as the reference. Multiple genome comparisons of plasmids from AHPND related isolates in Asian (A); Multiple genome comparisons of plasmids from AHPND related isolates in Mexico and Canada (B); Multiple genome comparisons of pVA-1 like plasmids from non-AHPND related isolates (C); the positions of genomic island

and *pirAB* were indicated in outer ring; Multiple genome comparisons of plasmids from other *Vibrio* sp. (D). Coloured rings indicate the sequence similarity between different genomes.
